# Medicinal Plants for Dermatological and Cosmetic Applications: Ethnobotanical Study from Northern Lithuania

**DOI:** 10.3390/plants15091389

**Published:** 2026-04-30

**Authors:** Daniele Urbonaite, Jurga Bernatoniene, Andrius Pranskunas, Zivile Pranskuniene

**Affiliations:** 1 Department of Drug Technology and Social Pharmacy, Lithuanian University of Health Sciences, LT-50162 Kaunas, Lithuania; 2 Institute of Pharmaceutical Technologies, Lithuanian University of Health Sciences, LT-50162 Kaunas, Lithuania; 3 Department of Intensive Care Medicine, Lithuanian University of Health Sciences, LT-50161 Kaunas, Lithuania

**Keywords:** cosmetics, ethnobotany, Lithuania, skin diseases, traditional medicine

## Abstract

Ethnopharmacological studies are growing in number in Europe; however, research on medicinal plants in Lithuania focusing on dermatological and cosmetic applications is still scarce. This study aimed to evaluate ethnobotanical heritage related to the treatment of skin diseases and cosmetic use in Northern Lithuania and to assess the compliance of traditional medicinal plant use indications with European Union herbal monographs. This study involved 36 participants aged 40 to 89. Data were collected using semi-structured interviews. This study documented 76 plant species belonging to 41 botanical families for the treatment of skin diseases and cosmetic purposes. This knowledge was primarily transmitted through family traditions, with 59.2% of respondents reporting that they acquired this knowledge from parents or grandparents. The medicinal plants most frequently mentioned for the treatment of skin diseases and cosmetic purposes were *Aloe vera* (L.) Burm. f. and *Plantago major* L. The most popular preparation method was topical application (32.4%) for treating skin diseases and decoction (38.5%) for cosmetic purposes. Plant-based raw materials were most often used to treat skin wounds (24.5%), as well as skin inflammation (16.3%) and burns (12.1%). For cosmetic purposes, the most frequently mentioned indication was dry skin (23.6% of plants). Of the 76 recorded plant species, 41 (53.9%) were not included in herbal monographs, and only 15 species (42.86%) were used in accordance with approved medical indications for skin diseases. Many plant species are used without European Medicines Agency-approved medical indications, relying solely on traditional and folk knowledge.

## 1. Introduction

Although contemporary modern medicine remains the primary choice for healthcare, a significant number of individuals still turn to traditional medicine. Public interest in the medicinal properties of plants is gradually increasing, accompanied by a growing demand for natural products [1]. This has also been confirmed by the World Health Organization, whose Traditional Medicine Strategy 2014–2023 aimed to promote the integration of traditional medicine practices, including herbal medicine, into health systems and to ensure their safe and effective use [2]. The increasing emphasis on preserving traditional knowledge and integrating it into modern healthcare has led to the expansion of ethnobotanical research in Lithuania. Consequently, the past decade has seen a marked rise in ethnobotanical and ethnopharmaceutical studies in the country [3,4,5,6].

Although Lithuania is a relatively small country, its geographical location results in the presence of diverse plant species and distinct local traditional knowledge. Geographically, Lithuania is divided into five ethnographic regions: Aukštaitija, Žemaitija, Dzūkija, Suvalkija, and Mažoji Lietuva (Minor Lithuania) [7]. Research has been conducted across various ethnographic regions to preserve ethnobotanical knowledge in Lithuania [5,8,9]. The majority of ethnopharmaceutical knowledge is still transmitted orally or recorded in various written sources, such as notes and manuscripts in Lithuanian. The preservation of ethnic knowledge has become especially relevant in the contemporary context, as it helps to maintain cultural heritage and enables the use of this knowledge in future research [6,8,9].

To the best of our knowledge, only one ethnopharmacological study has been conducted, analyzing medicinal plants used for skin diseases and cosmetic purposes during the COVID-19 period in Lithuania [6]. The study by Pranskuniene and colleagues [6] highlighted that while ethnobotanical research primarily targets skin conditions, many plant-based preparations used for therapeutic purposes are simultaneously applied for cosmetic use. Consequently, establishing a clear distinction between recorded preparations—namely cosmetics, cosmeceuticals, and pharmaceuticals intended for the treatment of skin diseases—remains highly challenging. However, the improper use of ethnopharmaceuticals for cosmetic purposes or for treating skin diseases may lead to aesthetic concerns as well as adverse health outcomes. Therefore, ethnopharmacological knowledge should be critically evaluated, grounded in rigorous scientific research, and aligned with the monographs of the European Medicines Agency.

Although there is an abundant supply of dermatological medications, their long-term use is often associated with adverse side effects. Consequently, medicinal plants and plant-based preparations have become increasingly relevant research subjects due to their milder and better-tolerated effects on the skin. Ethnobotanical studies in the field of dermatology are gaining greater significance, as they identify potential uses of traditional plants in the treatment of skin diseases [10].

The heightened consumerism observed in contemporary society, together with the substantial expansion of the beauty industry, has resulted in increased consumption of cosmetic products. The cosmetics sector is thriving, frequently introducing new products whose constituent ingredients and safety profiles are not always subject to rigorous pre-market assessment. Clinical and toxicological evaluations of chemical components in cosmetics are often conducted only after several years of market presence. Consequently, cases arise in which products that have been widely used are subsequently determined to pose health hazards and are withdrawn from the market [11,12,13,14].

Alongside the continuous growth of the cosmetics industry, the availability of natural cosmetic products has also increased. An expanding number of consumers are considering the potential adverse effects of synthetic substances on skin health; consequently, natural cosmetics are becoming increasingly valued. These products are primarily composed of plant-derived ingredients—such as oils, natural waxes and fats, and botanical extracts—and contain minimal synthetic additives [15]. Most plants exert complex, multifaceted effects, which are considered advantageous in the field of dermatology. However, further clinical and technological research is necessary to ensure the efficacy and safety of plant-based active compounds in cosmetic formulations [16]. In response to consumer expectations, modern mass-production cosmetics corporations are increasingly drawing inspiration for new product development from ethnobotanical sources. For example, tamanu oil has been traditionally employed in Polynesia for the treatment of burns, skin fissures, and eczema for several centuries. In the contemporary cosmetics market, tamanu oil is extensively applied and is associated with hundreds of patents, now held by major cosmetics industry companies [17].

The growing demand for natural ingredients is driving the increase in scientific research. The cosmetics and pharmaceutical industries are seeking innovative methods to preserve the activity of plant-derived ingredients efficiently and safely, for example, via nanoparticles or liposomal encapsulation. Furthermore, research has identified biologically active compounds within plant extracts and explored their applications in skin care [18,19,20]. Ethnobotanical research is particularly important in this field, as the accumulated traditional knowledge and experience can aid in identifying potential plants for skin health and care [16]. This study aimed to assess the surviving ethnobotanical knowledge in Northern Lithuania related to dermatological and cosmetic applications, emphasizing potential uses and aspects of safe utilization.

## 2. Results and Discussion

### 2.1. Study Participants

A total of 36 respondents participated in this study conducted in Šiauliai County, the majority of whom were women (32), with men accounting for only 4 respondents. This female-dominated gender distribution is a common phenomenon in ethnobotanical research in Lithuania. Traditionally, ethnobotanical knowledge in Lithuania is generationally passed down through the female lineage [3,8]. Another important factor observed during this study was the sense of community among women and their more active sharing of knowledge. Women were more likely than men to share practical information regarding the use of natural substances. Female respondents often participated in meetings where they exchanged collected plant materials, homemade products, and discussed skin health and beauty, topics that are primarily of interest to women. The age range of respondents was from 40 to 89 years, with the main group being between 60 and 89 years old, accounting for 78% of all participants.

When collecting data about the respondents, it was important to consider not only their current but also their native and previous places of residence. This information facilitated the evaluation of the origins of traditional knowledge. All participants in this study had a sedentary lifestyle. Several respondents mentioned that in their youth, they relocated elsewhere solely for educational purposes but eventually returned; thus, the collected data reflect the traditional knowledge specific to the Šiauliai County region. This study included respondents from 21 different localities within Šiauliai County. Of these, six were classified as cities, 10 as towns, and the remaining 5 as rural villages.

It was important during this study to determine respondents’ educational backgrounds and the sources of their ethnoknowledge. Respondents with higher, advanced, and secondary education were evenly distributed, each constituting 27.8% of all participants. Vocational education was held by 17.8% of respondents, while only one respondent (2.8%) had primary education. Those with higher education included agronomists, nurses, veterinarians, and zootechnicians. These professionals had been closely associated with agriculture and animal husbandry, and ethnobotanical knowledge had accompanied them since their youth. However, this study also included respondents not directly connected to agriculture, such as representatives from mathematics, economics, journalism, and educational fields. Therefore, the professional experience among participants was diverse and not limited to agricultural activities.

Since the respondents were generally educated individuals, it was important to assess the sources of their ethnobotanical knowledge. The most frequently cited sources were parents and grandparents, accounting for 59.2% of all mentioned knowledge sources. Given the previously reported age distribution of respondents, it is unsurprising that the transmission of ethnobotanical knowledge occurred in the traditional, oral manner, passed down generationally. Neighbors and acquaintances represented the second most common source, comprising 16.3% of responses, while books and newspapers accounted for 12.2%. In contrast, various forms of media (radio, television, and the internet) were among the least frequently mentioned sources of information, constituting only 6.1% of all responses.

### 2.2. Plant-Based Raw Materials for Skin Health and Beauty

This study documented 76 plant species belonging to 41 botanical families used for the treatment of skin diseases and cosmetic purposes. Table S1 (Supplementary Materials) presents all medicinal plants used for dermatological and cosmetic applications.

Among all cited plant families, *Asteraceae* was mentioned most frequently, accounting for 19.42% of the total citation frequency. Respondents identified 14 medicinal plant species belonging to this family. The *Lamiaceae* family ranked second in citation frequency (6.75%), accounting for 10 medicinal plant species. These results are consistent with data from ethnobotanical studies regarding dermatological applications conducted in Lithuania and other European countries [6,21,22].

According to the study results, the most culturally significant plants for skin health and beauty were *Aloe vera* (L.) Burm. f. and *Plantago major* L., each with a citation frequency of 97.2%, corresponding to 35 out of 36 respondents in this study. Among the most commonly used plants for skin disorders, *Plantago major* L. coincided with the results of Norwegian [23] and Greek studies [22]; however, it was not present in a study conducted in Romania and other Eastern European countries [21], where the most popular plants used for skin diseases corresponded to the other most popular plants in our study: *Matricaria recutita* L., *Chelidonium majus* L., *Calendula officinalis* L., and *Urtica dioica* L.

**Figure 1:**
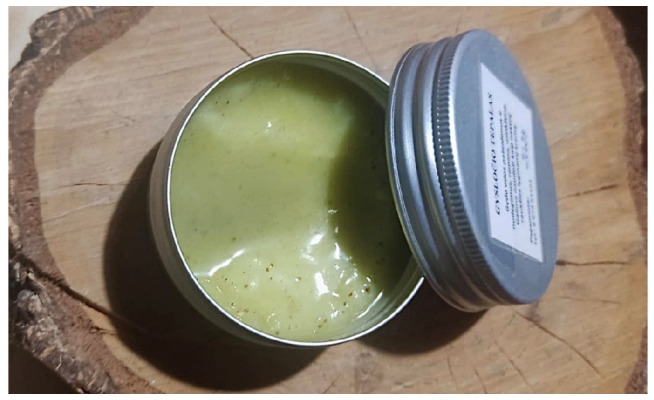
Homemade ointment prepared from *Plantago major* L. and pork fat by a participant aged 51 years. Image was taken on 22 August 2025 in Šiauliai.

*Plantago major* L., locally known as *trauklapis*, was used to treat superficial skin bruises. Respondents indicated that *Aloe vera* (L.) Burm. f. and *Plantago major* L. can be applied directly to the skin. *Plantago major* L. leaves should be washed, and aloe leaves should be cut open, with the inner part placed on the wound. In addition, respondents reported preparing and using an ointment derived from *Plantago major* L. leaves at home (Figure 1), used for bruises and wound healing.

The ointment is made from *Plantago major* L. raw material and pork fat. According to the recipe provided, two handfuls of chopped fresh plant (the whole plant with leaves, roots, and flowers) are mixed with 500 g of pork fat, placed in a glass container, and heated over low heat until the mixture acquires a bright green color. It is left overnight, heated the next day, strained through a linen cloth, and poured into glass containers. The production process involves many abstract measurements (only the amount of pork fat is accurate): the medicinal plant raw material is measured in handfuls, the degree of grinding is achieved individually, the mixture is heated until it acquires a bright green color, and no time is specified. Figure 1 shows the intensity of the green color, which is determined differently. The ointment is used for bruises and in cosmetics as a skin softener. Making this ointment requires skill and time, despite the most popular preparation method—topical application—which does not require any additives or effort. Pork fat has been used as a base for ointments for a long time in Lithuanian traditional medicine; nevertheless, the study participants noted that its specific smell complicates its use, especially for cosmetic purposes.

Numerous studies worldwide have acknowledged the pharmacological effects of *Plantago major* L. [24]. Moreover, numerous in vitro and in vivo studies have already investigated the wound-healing properties of *Plantago major* L. [25], which have long been used in traditional folk medicine in Lithuania [26]. The local plant name *trauklapis* is derived from the words *traukti* (to draw) and *lapas* (leaf); as explained by respondents, this results in “*trauklapis karštį traukia*” (*trauklapis* draws out heat), reflecting its traditional indications for use. However, further research is still needed to fully elucidate the precise mechanisms and activities of the primary bioactive compounds responsible for treating specific diseases. It has been reported that *Plantago major* L. has antimicrobial, antidiabetic, antispasmodic, antiviral, anti-inflammatory, and wound-healing properties, and it includes a wide range of bioactive substances, including phenolic compounds, flavonoids, terpenoids, iridoid glycosides, alkaloids, fatty acids, and polysaccharides [25]. Despite its exclusive external use in this study, it can cause side effects, such as allergic reactions [27].

*Aloe vera* (L.) Burm. f., locally known as *alijošius*, also has a long-standing tradition of use in Lithuanian folk medicine [26], and these traditions persist to the present day. Respondents in this study used the inner part of the aloe leaf for treating various wounds, burns, eczema, and dermatitis, as well as for dry skin. The use of aloe for skin health and beauty is still being investigated, including at the cellular level. It contains approximately 75 biologically active substances, including anthraquinones that inhibit cyclooxygenase activity, flavonoids, and saponins, and its antimicrobial properties have been shown to accelerate wound healing and stimulate collagen formation [28]. Nonetheless, despite its frequent use, this plant should not be considered devoid of potential toxic effects when used excessively. Ingestion of *Aloe vera* (L.) Burm. f. is associated with diarrhea, electrolyte imbalance, kidney dysfunction, and conventional drug interactions; episodes of contact dermatitis, erythema, and phototoxicity have been reported from topical applications [29,30].

Another frequently cited medicinal plant was *Matricaria recutita* L., with a citation frequency of 94.4%. Respondents identified seven different indications. Due to its anti-inflammatory properties, it was used to reduce various skin inflammations, alleviate redness and irritation, and treat burns and wounds. Linguistic studies of Lithuanian ethnobotanical taxa show that the local name *ramunė* belongs to the family of *rimti*, meaning to become quiet, calm [31]. Chamomile flowers were prepared and used as compresses, infusions, and hydrosols. The antioxidant activity of *Matricaria recutita* L. is mainly attributed to flavonoids and hydroxycinnamic acids. Numerous studies have demonstrated this plant’s anti-inflammatory properties and its potential applications in the treatment of skin diseases [32,33].

*Chelidonium majus* L. was also widely used by the inhabitants of Šiauliai County (86.1%). Respondents specifically highlighted the juice obtained directly from the stem of this plant. This “liquid,” as referred to by respondents, was [26] and continues [6] to be widely used to remove warts. The local name *karpažolė* further confirms the plant’s purpose, being derived from the words *karpa* (wart) and *žolė* (herb). Similar to the previously described plants (except *Plantago* ointment), this plant does not require any special preparation and is topically applied. This method is convenient and preserves the active compounds by avoiding their loss during processing. For comparison, *Chelidonium majus* L. is reported to be used in the treatment of psoriasis, impetigo, and tinea in Romania, but not in other European countries [21]. Alkaloids are the main active constituents of this plant. However, the potential hepatotoxicity of *Chelidonium majus* L. has also been confirmed in several reports from European countries [34]. However, in our study, this plant was used exclusively for external applications, which reduces the likelihood of toxicity. The concentrations of alkaloids in this plant differ according to the plant parts and growth conditions, but they generally have high medicinal value. Its potential toxicity risks must also be addressed [35].

*Calendula officinalis* L. was mentioned by 30 respondents. Decoctions, infusions, compresses, hydrosols, and ointments were prepared from its flowers. All methods of preparation were used to reduce local skin inflammation, treat wounds and burns, and alleviate dermatological conditions such as dermatitis and psoriasis. *Calendula officinalis* L. contains bioactive phytochemicals, such as flavonoids, triterpenoids, glycosides, saponins, carotenoids, and quinines, which are responsible for the plant’s anti-inflammatory, anticancer, wound-healing, and antioxidant properties. In Lithuanian folk medicine, this plant has traditionally been used for wound and burn treatment, based on sources of traditional knowledge transmission [26,36]. In clinical studies, due to its anti-inflammatory effects, it has been investigated for its potential role in promoting wound healing [37], including in the context of contemporary threats such as COVID-19 [38].

*Urtica dioica* L. was cited equally often, but respondents used it exclusively for cosmetic purposes, specifically for haircare. Decoctions were prepared from the roots and leaves, which were used as a hair wash to strengthen the hair, prevent hair loss, and eliminate dandruff. Although numerous studies have substantiated several pharmacological effects [39], in Lithuanian folk medicine, nettle decoction is specifically regarded as a homemade remedy for preventing hair loss, rather than for direct skincare or treatment [36]. The antioxidant effects of *Urtica dioica* L. extracts, as evidenced by the presence of effective antioxidant compounds, make them suitable for cosmetic formulations; their anti-inflammatory properties manage skin conditions and promote skin repair; and their antimicrobial activity may be beneficial in cosmetic formulations for product preservation. However, antimicrobial activity can cause microbiota imbalance [39].

### 2.3. Medicinal Plant Parts and Their Preparation Methods for Dermatological and Cosmetic Applications

**Figure 2:**
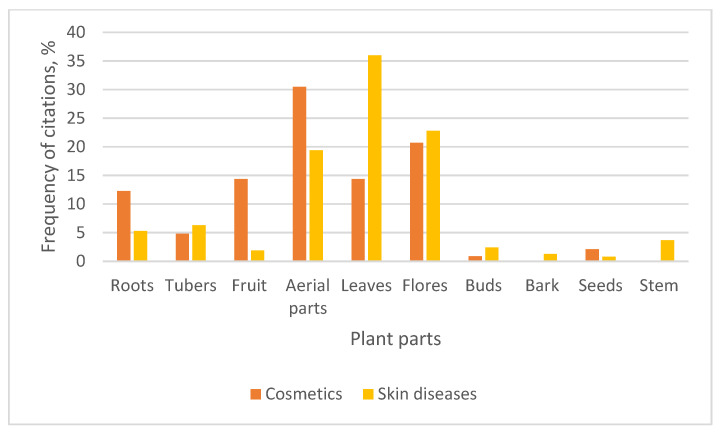
Plant parts used for cosmetic purposes and for skin diseases.

In this study, respondents identified specific parts of plants that were used for treating skin diseases or for cosmetic purposes. The distribution of these plant parts is presented in Figure 2.

Aerial parts and leaves dominated overall usage, though in slightly different ways across the two categories. Aerial parts were the most frequently cited for cosmetic purposes (30.5% of all mentions), suggesting that above-ground portions—likely including stems, leaves, and flowers collectively—are preferred for formulations aimed at beauty, skincare maintenance, or enhancement. This may be due to their accessibility, higher yield, and richness in aromatic or bioactive compounds useful in cosmetic preparations.

In contrast, leaves showed the highest citation for treating skin diseases (36% of all mentioned plant parts), making them the most important plant part in medicinal dermatological applications. Likewise, Tsioutsiou, E. E. and colleagues in Greece [22] and Ijeabuonwu, A. M. and colleagues in Norway [23] found that leaves were the most frequently used either directly or to prepare remedies against skin ailments. Leaves are often rich in secondary metabolites such as flavonoids, tannins, and antimicrobial compounds that are effective in treating infections, inflammation, and other skin conditions.

Flowers also showed relatively high usage in both categories, with a slightly higher emphasis on skin disease treatment (22.8% of all mentions). This suggests that flowers may contain compounds beneficial for soothing, anti-inflammatory, or antiseptic purposes, while still being valued for their aesthetic or fragrance properties in cosmetics (20.7% of cases), as mentioned in the study by Pranskuniene, Z. and colleagues in Lithuania [6].

Roots and fruits displayed moderate use, but with a noticeable preference for cosmetics over skin disease treatment. Roots, in particular, may be valued for their nourishing or rejuvenating properties in traditional cosmetic formulations, whereas their medicinal use for skin conditions appeared less prominent in this dataset.

**Figure 3:**
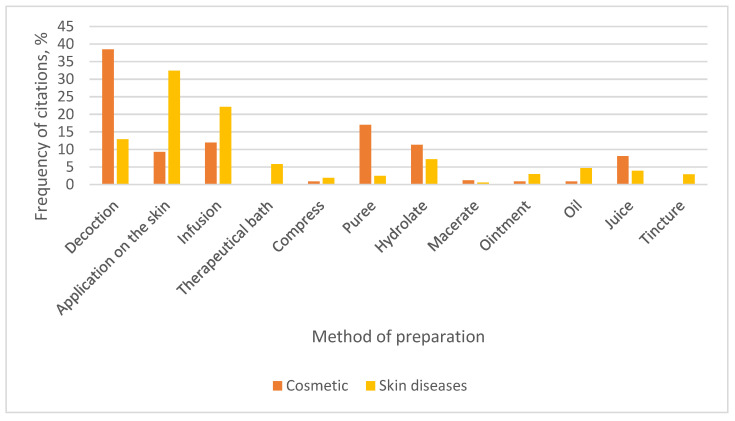
Methods of preparation.

The preparation methods for these plant-based raw materials are presented in Figure 3.

Decoction was the dominant preparation method for cosmetic purposes (38.5% of all mentions), with a markedly higher percentage compared with its use in treating skin diseases (12.9%). This suggests that water-based extraction via boiling is culturally preferred for cosmetic applications, possibly because it efficiently extracts hydrophilic compounds such as phenolics, flavonoids, and tannins, which are known for their antioxidant and skin-soothing properties. The lower but notable use of decoctions in skin disease treatment indicates that they are also valued therapeutically.

Respondents also commonly reported the use of plants for local skin applications (32.4% for skin diseases and 9.3% for cosmetic purposes). In cases of skin diseases, the plant material was used fresh, most often applied directly as leaves onto the skin. This aligns with an ethnobotanical study in Greece for skin diseases [22], where the most frequent method was also the topical application of the fresh plant part (35.3%). Direct topical application allows bioactive compounds to act locally on infections and is an important part of quick first aid. The high percentage implies a strong reliance on fresh plant material, pastes, or minimally processed forms, preserving volatile and heat-sensitive compounds that might otherwise degrade during boiling. The most frequently cited plants for such applications were *Aloe vera* (L.) Burm. f., *Plantago major* L., *Arctium lappa* L., and *Brassica oleracea* L. For example, the aloe leaf is cut and split longitudinally and then placed on the affected skin area. According to respondents, the leaf of *Arctium lappa* L. should be applied with the smooth side facing the skin (“the side that faces the sun”).

**Figure 4:**
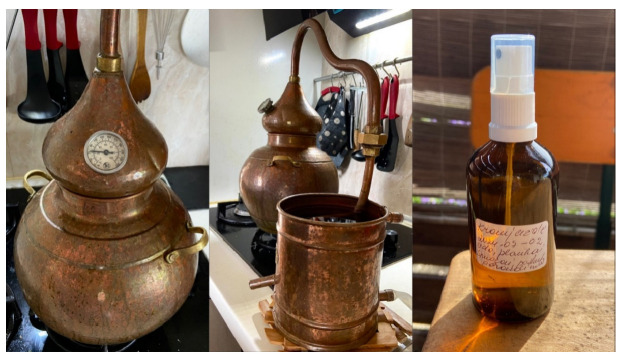
Hydrolate production in a domestic environment: alembic apparatus and the final labeled product (hydrolate). Participant aged 59 years. Images were taken on 9 July 2025 in Kražiai.

Another preparation method for plant-based raw materials mentioned by respondents was hydrolates (7.2% for skin disease treatment and 11.3% for cosmetic purposes). Hydrolates are prepared at home from plant herbs (aerial parts) or flowers, using a special distillation apparatus called an alembic. Several respondents indicated that they own an alembic and utilize it for hydrolate production at home (Figure 4).

Hydrolates, as a preparation form, were a notable finding of this study, indicating a shift in contemporary ethnopharmacological research. This use mode was not mentioned in the subject matter examined in studies conducted in Lithuania [6] nor in recent ethnobotanical research conducted in other countries [22,23]. Simple topical application (for example, when a plant leaf is picked and placed directly on a wound) requires only knowledge, making the method “readily accessible” and quick. This readily accessible and rapid preparation method is also characteristic of homemade preparations for skin diseases in other countries [22] and indicates that ease of use and accessibility are important factors for rapid first-aid interventions. In contrast, the preparation of hydrosols at home requires the combination of traditional folk medicine knowledge with modern opportunities to access specialized equipment and acquire the skills necessary to operate it.

**Figure 5:**
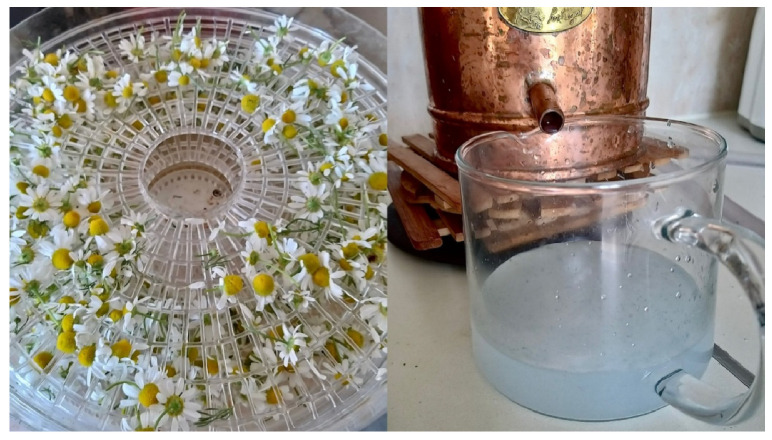
Hydrolate production (demonstration) from *Matricaria recutita* L. flowers. Participant aged 59 years. Images were taken on 9 July 2025, in Kražiai.

Respondents store their homemade hydrolates in dark glass bottles, either with a spray nozzle or without. Each bottle is labeled with the preparation date and intended use. Lavender hydrolate was particularly popular among respondents, being used as a facial toner for dry skin in cosmetic applications and for reducing inflammation in cases of skin diseases. According to respondents, hydrolate of peppermint (*Mentha piperita* L.) aerial parts is used to care for oily, acne-prone facial skin and as a facial toner. Rose petal hydrolate is chosen for maintaining dry or acne-prone facial skin. Chamomile (*Matricaria recutita* L.) flower hydrolate is employed as an anti-inflammatory agent. The raw material is collected and dried at home, after which hydrolate production is undertaken (Figure 5).

Respondents also indicated that they prepare a paste (2.5% for skin disease treatment and 17% for cosmetic purposes), which involved crushing fruits or herbs and applying the resulting mass directly to the skin. For cosmetic purposes, the paste was most commonly used as a facial mask; for example, parsley (*Petroselinum crispum* (Mill.) Fuss), wild strawberry (*Fragaria vesca* L.), and commercial strawberry (*Fragaria × ananassa*) were used to whiten facial skin and reduce hyperpigmentation.

### 2.4. Conditions for Storage and Collection of Medicinal Plant Raw Materials

All 36 respondents indicated that natural conditions are important to them when collecting medicinal raw materials. In total, 94.4% of respondents stated that they consider the time of day during collection. Some reported gathering herbs early in the morning, but emphasized that the plants should not be covered with dew. Others mentioned collecting plants at midday. In both cases, the main consideration was that the plants be harvested dry. Respondents who produce hydrolates additionally pay attention to the intensity of fragrance in flowering plants. For example, peony flowers are preferably collected in the evening for hydrosol preparation because their aroma is more intense at that time, whereas rose petals are best collected early in the morning, as respondents noted that hydrolates made from such petals have a stronger scent.

Most respondents (88.9%) considered seasonality important. Respondents reported collecting different parts of the plant at different times of the year. For example, roots are harvested in spring, before the plant has flowered, or in autumn, after flowering, when active compounds are accumulated in the roots. Flowers are typically collected during the plant’s bloom, most often at the beginning of summer, while the aerial parts are gathered at the onset of flowering. Fruits, according to respondents, should be collected when fully ripe.

Several respondents also emphasized the traditional belief that plants are best collected before *Joninės*, also known as *Rasos* or *Kupolinės* (one of the oldest Lithuanian celebrations held at the summer solstice, when the day is longest, and the night is shortest).

Weather conditions were considered by 86.1% of all study participants. All of them indicated that medicinal plants should be harvested during dry and sunny weather, avoiding collection after rain or when covered with dew.

A quarter of respondents (25%) reported paying attention to the positions of celestial bodies, such as moon phases. According to respondents, the lunar cycle can influence sap movement through the stem, seed germination, tuber maturation, and root strength. During the new moon (*jaunatis*), when the crescent moon is visible, it is recommended to collect shoots, buds, and flowers. In the waxing moon (*priešpilnis*), the above-ground parts, leaves, stems, and flowers are best collected. During the full moon (*pilnatis*), respondents considered it the optimal time to harvest flowers and fruits. As the moon wanes (*delčia*), respondents collect roots and tubers. The positioning of celestial bodies was relevant to one in four respondents, and these principles correspond to those described in Lithuanian traditional folk medicine [26].

**Figure 6:**
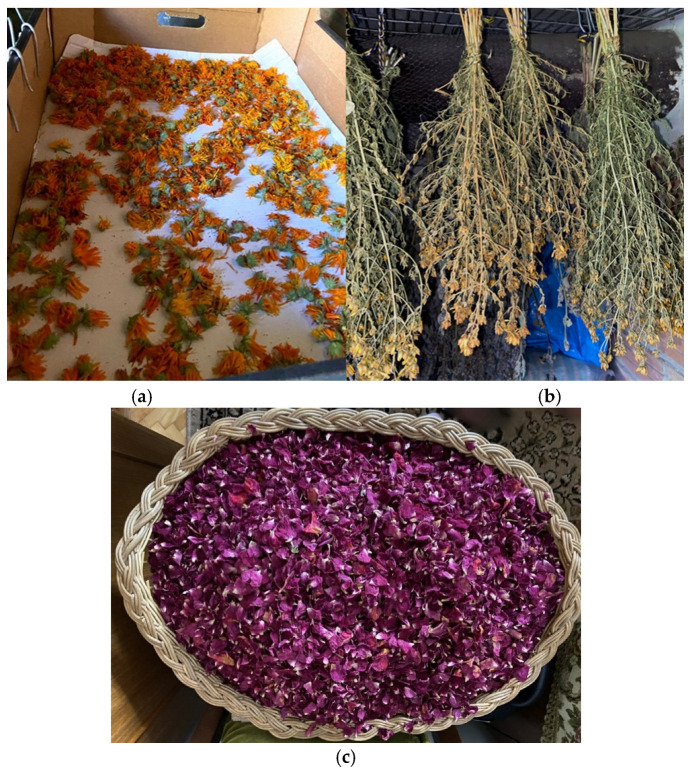
Drying of medicinal plants: (**a**) *Calendula officinalis* L.; (**b**) *Hypericum perforatum* L.; (**c**) storage of *Rosa damascena* Mill. petals by a participant aged 82 years. Images were taken on 24 July 2025 in Tytuvėnai.

During the survey, respondents also indicated that they pay attention to the plant’s habitat. Plants should be collected in natural environments, away from roads and larger settlements, to avoid dust and pollution. Furthermore, the soil should not be fertilized. Proper drying and storage were essential for respondents to preserve medicinal herbs and their beneficial compounds. Medicinal plants are dried in cool, well-ventilated spaces that are protected from direct sunlight. Some respondents said that herbs are best dried individually, spread out in a thin layer. However, some respondents dry herbs in a bundled form (Figure 6).

During this study, respondents also specified the conditions under which they store dried medicinal herbs: away from direct sunlight; in a dark place; using cloth, paper, or fabric bags, as well as glass containers. However, respondents do not seal the lids of glass containers tightly to prevent moisture accumulation and mold formation.

### 2.5. Medicinal Plants for Dermatological Applications

**Figure 7:**
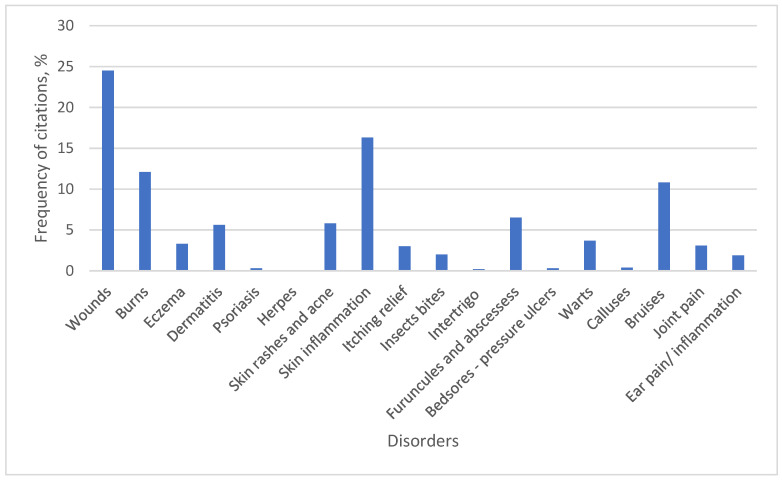
Medicinal plants for dermatological applications.

Figure 7 illustrates the disorders identified during this study that are treated with homemade herbal preparations.

The most frequently mentioned skin disease indication among respondents was wounds (24.5%). Respondents referred to various types of wounds, including cuts, trophic ulcers, poorly healing wounds, and minor abrasions. Wounds are a primary skin ailment according to this and other ethnobotanical studies [40]. Most study participants, i.e., 35 out of 36 respondents (97.2%), reported using *Aloe vera* (L.) Burm. f. for wound treatment. *Calendula officinalis* L. was also frequently mentioned, cited by 25 respondents (69.4%). Respondents emphasized that aloe was previously found in nearly every household and used to address various health issues, ranging from wounds to internal diseases. These traditional practices are corroborated by scientific studies, which demonstrate that the polysaccharides (such as acemannan) present in aloe promote skin regeneration and wound healing [16].

Skin inflammation was the second most frequently cited indication (16.3%), described by respondents as skin redness, swelling, or scaling of unclear origin. Most respondents (85.7%) indicated that *Calendula officinalis* L. and *Matricaria recutita* L. are traditionally used for treating skin inflammation. Infusions, compresses, and decoctions were prepared from their flowers, as well as hydrolates. Both medicinal plants are widely recognized for their anti-inflammatory properties [28].

Another popular indication for skin disease treatment was burns, accounting for 12.1% of all indications mentioned. Respondents selected previously cited plants, i.e., *Aloe vera* (L.) Burm. f. and *Calendula officinalis* L., for treating burns. Similar results have been obtained in ethnobotanical studies conducted in the Mediterranean region, where *Calendula officinalis* L. is traditionally used for wound and burn treatment [28].

Bruises were also among the most frequently mentioned indications (10.8%). For their treatment, respondents cited the use of *Plantago major* L., applying washed leaves directly to the bruised area. The leaves of *Arctium lappa* L. were also mentioned; these were blanched with hot water before use to soften them.

Skin rashes and acne accounted for 5.8% of all mentions. For these indications, respondents chose decoctions of oak (*Quercus robur* L.) bark and birch (*Betula pendula* Roth.) buds, which were used to wash and cleanse the skin. Specific components of these trees have potential for dermal and cosmetic applications and act more gently than aggressive chemical drugs, particularly for teenagers with acne [41].

### 2.6. Medicinal Plants for Cosmetic Applications

**Figure 8:**
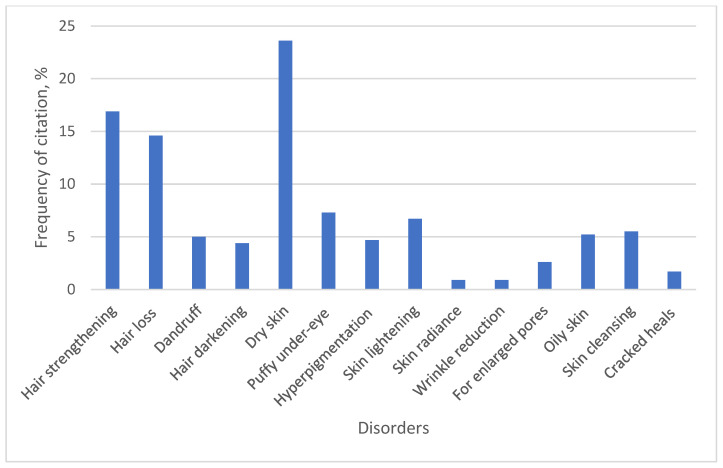
Medicinal plants for cosmetic applications.

Medicinal plants were used for a total of 14 cosmetic indications. The results are presented in Figure 8.

Respondents identified four indications related to hair care: hair strengthening (16.9%), hair loss prevention (14.6%), dandruff control (5%), and hair darkening (4.4%). A study in Norway also showed attention to hair cosmetics [23]. Collectively, these indications accounted for 40.9% of all citation frequencies for cosmetic purposes. Respondents rinsed their hair with decoctions made from the aerial parts of *Urtica dioica* L. to strengthen their hair, add shine, prevent hair loss, and avoid dandruff. Additionally, they stated that rinsing hair with *Acorus calamus* L. root decoction reduces hair loss and strengthens hair against breakage. For hair darkening, respondents used oak (*Quercus robur* L.) bark decoctions.

The remaining indications (57.1%) were related to facial skincare. Care for dry skin (23.6%) was the most frequently cited cosmetic indication. Skin hydration is the main target of ethnobotanical preparations for cosmetic purposes in studies conducted in Norway [23], Italy [42], and Lithuania [6]. For dry skin, most respondents used *Aloe vera* (L.) Burm. f., mentioned by 77.7% of all respondents. Another plant chosen by respondents (47.2%) for dry skin care was *Cucumis sativus* L. For facial skincare, respondents indicated the use of hydrosols made from lavender, rose, and peony petals, as well as decoctions of flax seeds and oats.

Another cosmetic indication was skin lightening, which accounted for 6.7% of all mentions. To lighten facial skin, respondents indicated the use of *Betula pendula* Roth. bud decoction. In addition, a paste made from the aerial parts of *Petroselinum crispum* (Mill.) Fuss was applied as a facial mask.

Skin impurities accounted for 5.5% of all cosmetic indications cited. The most popular plant for this purpose was *Saponaria officinalis* L., often referred to by respondents as “muiliažolė,” cited by 52.7% of all respondents. According to respondents, rubbing the herb of *Saponaria officinalis* L. between the hands and mixing it with water produces foam that effectively cleanses body impurities.

### 2.7. Home-Made Preparations and European Medicines Agency Monographs

Contemporary ethnopharmaceutical studies conducted in Lithuania demonstrate that ethnopharmaceutical practices, particularly in small towns and rural areas, remain an important component of healthcare [1,3,5,6,8,9]. A segment of the population still mistakenly believes that medicinal plants, fungi, or other substances of natural origin cannot cause adverse effects. Conversely, scientific research emphasizes that natural raw materials that are incorrectly collected, prepared, and used for self-medication may pose risks to consumer health [43]. The World Health Organization notes that the use of herbal preparations as complementary and alternative medicine has significantly increased over recent decades [44], highlighting the need to strengthen pharmacological surveillance and consumer education. These findings underscore the need for a more comprehensive evaluation of ethnopharmaceutical knowledge, its safety, and its efficacy, aiming to integrate it into modern medicine. A comparison of our study data with European Medicines Agency monographs is presented in Table S1 (Supplementary Materials).

The study results showed that out of 76 different plant species mentioned by respondents, 35 (46.1%) are included in the European Medicines Agency (EMA) monographs (Table 1).

The remaining 41 medicinal plants were used by respondents based on their own accumulated knowledge, which, as this study showed, is predominantly passed down generationally. A total of 59.6% of respondents reported that their knowledge about medicinal plant use was acquired from parents or grandparents. Of the 35 medicinal plants included in the EMA monographs, only 15 (42.9%) matched the indications provided by the EMA. This indicates that the remaining 20 (57.1%) medicinal plants were either used outside of the EMA recommendations or their uses only partially corresponded to those outlined in the monographs.

For example, respondents used the hydrolate of rose (*Rosa damascena* Mill.) petals for cosmetic purposes, such as for dry and acne-prone skin care. However, the EMA monograph lists rose petals as a remedy for minor skin inflammation, and, thus, this use did not align with the monograph indications. Similarly, the EMA monograph states that birch leaves (*Betulae folium*) are used for mild urinary tract disorders as a diuretic. Respondents, however, reported using birch leaf decoction externally for facial skin whitening and hair thickening, as well as birch buds for treating skin rashes and shoots for wound healing. The EMA monograph for *Aloe vera* (L.) Burm. f. indicates applying dried aloe leaf juice (*Aloes folii succus siccatus*) for oral use as a laxative, whereas respondents used the inner leaf for various external skin disorders and cosmetic purposes.

Although only 15 medicinal plants matched the EMA monograph indications, these plants were cited by a large proportion of respondents. For instance, *Matricaria recutita* L. was among the most frequently mentioned plants, used by 30 respondents (83.3%) for treating skin inflammation and by 14 (38.9%) for wound healing. Both indications are consistent with EMA recommendations. Other frequently cited medicinal plants (mentioned more than 10 times) whose use aligned with EMA indications included *Calendula officinalis* L., *Achillea millefolium* L., *Arctium lappa* L., *Arnica montana* L., *Hypericum perforatum* L., *Quercus robur* L., *Salvia officinalis* L., and *Symphytum officinale* L.

During the survey, respondents were also asked whether they consult a physician or pharmacist about using medicinal herbs. Of the thirty-six respondents, only three indicated that they seek advice from a pharmacist, while none consulted a physician about herbal use. Some participants stated that they do not wish to waste the time of physicians or pharmacists, while others believed that modern medical professionals are not interested in herbal medicine. Most respondents expressed confidence in their own knowledge; thus, they did not feel the need for consultation. Data from other studies conducted in Lithuania also show that consultation with healthcare professionals regarding the use of homemade preparations is uncommon [5], which raises safety concerns.

This situation changed during the COVID-19 pandemic, when, specifically regarding ethnobotanical remedies used for skin diseases and cosmetic purposes, every other respondent reported consulting a pharmacist [6]. Thus, in critical situations, the pharmacist remains the primary healthcare professional who bridges traditional medicine and evidence-based phytotherapy knowledge.

## 3. Materials and Methods

### 3.1. Study Procedure

First, efforts were made to become familiar with the study area and its residents and to collect information about the participants, who were local inhabitants. Most respondents were elderly individuals with practical experience in using plant-based remedies for self-treatment. Several respondents were engaged in the production and sale of homemade preparations, such as hydrolates.

Participants were selected using the “snowball” sampling method, whereby one respondent recommends another member of the target group who is willing and able to share their knowledge regarding the use of natural substances for the treatment of skin diseases and cosmetic purposes. Herbalists living in Northern Lithuania (Šiauliai County) maintain close connections and form a community that exchanges practices, plant raw materials, and homemade products. Recommendations from respondents often overlapped, which strengthened the reliability of the collected knowledge and affirmed the authority of respondents within local communities (when multiple participants recommended the same individual).

Additionally, during the course of this study, it was revealed that some respondents had an interest in medicinal plants, as well as having actively worked in agriculture throughout their lives, with educational backgrounds in agriculture and animal husbandry.

**Figure 9:**
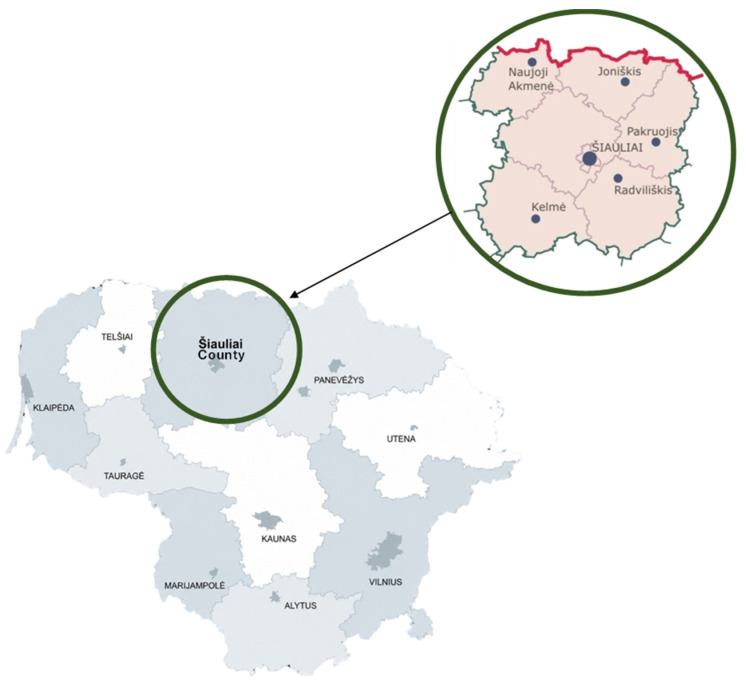
Study area.

The field study was conducted from June to September 2025 in Northern Lithuania, Šiauliai County (Figure 9). In total, 36 respondents were surveyed in Šiauliai County, of whom 32 were women, and 4 were men.

This study was conducted per the Code of Ethics of the International Society of Ethnobiology [45]. This study was approved by the Bioethics Committee of the Lithuanian University of Health Sciences (No. 2025-BEC2-0281). Interview times and locations were arranged in advance, and participants were informed about the study topic so that they could recall or review their notes. Respondents were interviewed using a semi-structured interview method, conducted during in-person meetings. Before the interview, participants signed an informed consent form. With respondents’ permission, the interviews were recorded using an audio device, which ensured the accuracy and completeness of the information collected.

The semi-structured interview questionnaire consisted of 17 questions, with both closed and open-ended examples. Data were collected regarding the plants that respondents collected and cultivated, their collection and storage conditions, and demographic information (age, gender, and education). Details on the previous and current place of residence were also recorded to ensure that the study results accurately reflected the ethnobotanical knowledge specific to Šiauliai County. This constituted the first stage of this study.

The second stage included questions about the use of natural substances, plants, and medicinal raw materials; their preparation methods; and indications for use. These questions were open-ended, allowing respondents to freely share their knowledge and personal experience. During this stage, respondents demonstrated their homemade products (e.g., hydrosols and ointments), identified their production sites, and described the processing steps.

The data on medicinal plants collected during this study were compared with the usage indications provided in the European Medicines Agency monographs [46]. These monographs provide assessments of the safety and efficacy of medicinal plants, their parts, and preparations. This comparison aimed to evaluate the extent to which the use indications recorded in this study corresponded to those described in EMA monographs. A significant proportion of medicinal plants were identified based on the names provided by respondents, as well as descriptions of their appearance and properties (using traditional floras and atlases of Lithuanian flora) [47,48]. Some of the plants were cultivated by respondents in their homes or home gardens, enabling visual identification during the interviews. Additionally, visits to natural habitats were conducted together with respondents. The database “World Flora Online” [49] was used for taxonomic identification, botanical nomenclature determination, and classification of plant families.

### 3.2. Study Area

Šiauliai County is one of Lithuania’s ten administrative regions. Figure 9 shows that the county is located in the northern part of the country. It borders Telšiai County to the west, the Republic of Latvia to the north, Tauragė County to the southwest, Kaunas County to the south, and Panevėžys County to the east. The area of Šiauliai County covers 8537 km^2^, representing approximately 13.1% of Lithuania’s territory. The county contains a total of 15 cities and 2619 rural settlements, with Šiauliai city serving as its administrative center. According to official data from 2021, Šiauliai County has a population of approximately 260,000 [50].

The Dubysa and Venta rivers, along with their tributaries, are the most significant rivers in Šiauliai County, while forests and woodlands cover 27.5% of the county’s territory. Additionally, Šiauliai County contains 37 lakes and over 100 ponds.

The Šiauliai region occupies a unique ethnographic position, as it is attributed to two ethnographic regions: Žemaitija and Aukštaitija. These regions’ boundaries are relative, so border areas may exhibit intertwined cultural traditions and dialects.

Šiauliai County, like all of Lithuania, belongs to the temperate transitional climate zone, situated between maritime and continental climate types, characterized by four distinct seasons [51].

Šiauliai County is characterized by fertile soils, extensive agricultural land, and a strong agricultural industry. According to 2022 data, the largest share of Lithuania’s agricultural production originated in Šiauliai County, accounting for one-fifth of the country’s total output. Moreover, the majority of this production consisted of crop farming products [7].

## 4. Conclusions

Medicinal plants remain an important part of everyday self-care for dermatological and cosmetic applications in Northern Lithuania. Many plant species are used without European Medicines Agency-approved medical indications, relying solely on traditional and folk knowledge. The results of this study emphasize the importance of collecting ethnoknowledge for dermatological and cosmetic applications and improving public education regarding the safe use of natural medicinal resources. Ethnobotanical studies specifically focused on dermatological applications are relatively scarce; however, the few studies that exist often overlook cosmetic indications, which are frequently interconnected and carry sociocultural, health-promoting, and heritage-related significance and could be an important direction for future research.

## Figures and Tables

**Table 1:** Study data comparison with EMA monographs.

Category	Status	Study Data (n)	Study Data (%)
EMA evaluation	Present	35	46.1
	Absent	41	53.9
Compliance with indications	Coincides	15	42.86
	Does not coincide	20	57.14

## Data Availability

The original contributions presented in this study are included in this article/the Supplementary Material; further inquiries can be directed to the corresponding author.

## Supplementary Materials

The following supporting information can be downloaded at https://www.mdpi.com/article/10.3390/plants15091389/s1. Table S1 Medicinal plants used to treat skin diseases and for cosmetic purposes in Northern Lithuania; File S1. Questionnaire.
