# Supplementary material for: Medicinal Plants for Dermatological and Cosmetic Applications: Ethnobotanical Study from Northern Lithuania
Source: Plants (Basel). 2026 Apr 30;15(9):1389. doi: 10.3390/plants15091389 (PMC13165265; doi:10.3390/plants15091389)
Supplement: Supplementary file 1 [file plants-15-01389-s001.zip › Table S1.pdf]

Family	Botanical name	Local plant name	Used for skin diseases – L Used in cosmetics - K	Infor - mants (N)	Use-reports (UR)	Part used	Method of preparation	Therapeutic and cosmetic uses	EMA evaluation
Acoraceae	*Acorus calamus* L.	Balinis ajeras	L<br>K	25	17<br>3<br>12<br>2<br>1<br>1	Roots<br>Aerial parts<br>Roots<br>Roots<br>Aerial parts<br>Aerial parts	Decoction<br>Hydrolate<br>Decoction<br>Decoction<br>Hydrolate<br>Hydrolate	For hair strengthening<br>From hair loss<br>For hair darkening<br>Wound healing<br>For intertrigo	
Amaranthaceae	*Beta vulgaris* L.	Burokas	K	2	2	Roots	Topical application (on the cheeks)	For skin radiance	
Amaryllidaceae	*Allium cepa* L.	Svogūnas<br>Cibulis	L<br>K	29	29<br><br>5	Tubers<br><br>Tubers	Topical application<br>Juice	To treat furuncles and abscesses<br>From hair loss	
Amaryllidaceae	*Allium sativum L.*	Česnakas	K	8	8	Tubers	Puree	From hair loss	EMA/HMPC/7685/2013 *Allii sativi bulbus;* Prevention of atherosclerosis*;* Common cold
Apiaceae	*Foeniculum vulgare* L.	Pankoliai	K	4	4	Fruits	Infusion (face rinse)	Skin rashes and acne	*EMEA/HMPC/372839/2016 Foeniculi dulcis fructus;* Cough and cold; Gastrointestinal tract disorders; Menstrual spasms

Apiaceae	*Petroselinum crispum* (Mill.) Fuss	Petražolė<br>Petruška	K<br>L	10	3<br><br>8<br><br>1<br>2	Aerial parts<br><br>Aerial parts<br><br>Aerial parts<br>Aerial parts	Puree<br><br>Puree<br><br>Puree<br>Compress	To reduce enlarged pores<br>For skin lightening<br>For wrinkles<br>To reduce skin inflammation	
Asparagaceae	*Polygonatum odoratum*	Baltašaknė	K	1	1	Roots	Topical application (on the cheeks)	For face radiance	
Asphodelaceae	*Aloe vera* Mill.	Alavijas<br>Alijošius	L<br>K	35	35<br>33<br>9<br>9<br>28	Leaves	Topical application	Wound healing<br>For burns<br>To treat eczema<br>To treat dermatitis<br>For dry skin	EMA/HMPC/625788/2015<br>*Aloes folii succus siccatus;* Short-term use in cases of occasional constipation
Asteraceae	*Achillea millefolium* L.	Kraujažolė	L	23	19<br><br><br>2<br>2	Leaves/ Aerial parts<br><br>Aerial parts<br>Flores	Topical application/ Decoction<br>Hydrolate<br>Macerate	Wound healing<br><br><br>Wound healing<br>Wound healing	EMA/HMPC/143949/2010<br>*Millefolii flos;* Gastrointestinal disorders; Loss of appetite; Skin disorders and minor wounds; Menstrual spasms<br>EMA/HMPC/376416/2019<br>*Millefolii herba;* Gastrointestinal disorders; Loss of appetite; Skin disorders and minor wounds; Menstrual spasms
Asteraceae	*Arctium lappa* L.	Varnalėša	L<br>K	16	16<br><br>3<br>3<br>4	Leaves<br><br>Leaves<br>Leaves<br>Roots	Topical application<br>Compress<br>Compress<br>Decoction	For bruises<br><br>To treat eczema<br>To treat dermatitis<br>To strengthen hair	EMA/HMPC/246763/2009<br>*Arctii radix; Urinary tract disorders;* Loss of appetite*;* Skin disorders, seborrheic skin
Asteraceae	*Arnica montana* L.	Arnika	L	13	10<br>3<br>3<br>2	Aerial parts<br>Aerial parts<br>Aerial part<br>Aerial parts	Tincture<br>Infusion<br>Infusion<br>Tincture	For bruises<br>Wound healing	EMA/HMPC/198793/2012

								To treat skin rashes<br>For furuncles and abscesses	*Arnicae flos; Bruises, sprains and localized muscular pain*
Asteraceae	*Artemisia absinthium* L.	Pelynas	L	1	1	Aerial parts	Topical application	Small wound healing	EMA/HMPC/751490/2016<br>*Absinthii herba;*<br>Gastrointesti-nal disorders; Loss of appetite
Asteraceae	*Bidens tripartita L.*	Lakišius	L	5	5	Aerial parts	Therapeutic bath	To treat skin rashes and acne	
Asteraceae	*Calendula officinalis*	Medetka<br>Medatka<br>Nadatka	L<br>K	30	26<br>4<br><br>21<br>4<br>20<br>16<br>16<br>1<br>1<br><br>1<br>1<br>4<br>5	Flores<br><br><br>Flores<br><br>Flores<br>Flores<br>Flores<br>Flores<br>Flores<br><br>Flores<br>Flores<br>Flores<br>Flores	Infusion<br>Hydrolate<br><br>Infusion<br>Hydrolate<br>Infusion<br>Infusion<br>Infusion<br>Hydrolate<br>Topical application<br>Puree<br>Infusion<br>Infusion<br>Hydrolate	To reduce skin inflammation (redness, swelling)<br>Wound healing<br><br>For burns<br>To treat eczema<br>To treat dermatitis<br>To treat bedsores<br>For wart treatment<br><br>For dark spots<br>To strengthen hair<br>For skin lightening<br>To treat skin rashes and acne	EMA/HMPC/437450/2017<br><br>*Calendulae officinalis* flos; skin disorders (sunburns) and minor wounds; Mouth and throat disorders
Asteraceae	*Centaurea cyanus L.*	Rugiagelė	K	2	1<br>2	Flores<br>Flores	Hydrolate<br>Hydrolate	For dry skin<br>For oily skin	
Asteraceae	*Cirsium arvense* (L.) Scop.	Dirvinis usnis	L	1	1<br>1<br>1	Aerial parts<br>Aerial parts<br>Aerial parts	Decoction<br>Decoction<br>Decoction	To treat eczema<br>For bruises<br>To reduce itching	
Asteraceae	*Echinacea purpurea* L. (Moench)	Ežiuolė	K	2	2	Roots	Decoction	To treat skin rashes and acne	EMA/HMPC/48704/2014<br>*Echinacea purpurea herb*a;<br>Skin disorders and minor wounds<br>EMA/HMPC/424583/2016

									Echinacea purpurea radix;<br>Common cold and cough;<br>For spots and pimples due to mild acne
Asteraceae	*Gnaphalium uliginosum* L.	Pelkinis pūkelis	L	1	1<br><br>1	Aerial parts<br><br>Aerial parts	Decoction<br><br>Decoction	To treat skin rashes and acne<br>Wound healing	
Asteraceae	*Helianthus tuberosus* L.	Topinambas<br>Bulvinė saulėgrąža	L	6	6<br><br>1<br><br>6	Leaves (young)<br>Aerial parts<br><br>Tubers	Therapeutic bath<br>Therapeutic bath<br>Puree	Wound healing<br><br>Wound healing<br><br>For burns	
Asteraceae	*Inula helenium* L.	Didysis debesylas	L	3	3<br>3	Leaves<br>Roots	Therapeutic bath<br>Decoction	To treat psoriasis	
Asteraceae	*Matricaria recutita* L.	Ramunėlė<br>Vaistinė Ramunė	L<br>K	34	26<br>4<br><br><br>10<br>4<br>7<br>4<br>17<br>14	Flores<br><br><br><br>Flores<br>Flores<br>Flores<br>Flores<br>Flores<br>Flores	Infusion<br>Hydrolate<br><br><br>Infusion<br>Hydrolate<br>Hydrolate<br>Infusion<br>Infusion<br>Infusion	To reduce skin inflammation (redness, swelling)<br>Wound healing<br>Wound healing<br>For burns<br>To treat dermatitis<br>Under-eye puffiness<br>To strengthen hair	EMA/HMPC/ 55843/2011<br>*Matricariae flos*;<br>Gastrointestinal disorders;<br>Common cold and cough;<br>Mouth and throat disorders;<br>Skin disorders and minor wounds
Asteraceae	*Taraxacum officinale L.*	Kiaulpienė	L<br>K	4	2<br><br>2<br>4	Flores<br><br>Flores<br>Flores	Macerate<br><br>Macerate<br>Macerate	For furuncles and abscesses<br>For burns<br>For dry skin	EMA/HMPC/579636/2008<br>*Taraxaci folium*; Urinary tract disorders<br>EMA/HMPC/212895/2008<br>*Taraxaci officinalis radix cum herba;* Urinary tract disorders; Gastrointestinal disorders; Loss of appetite
Betulaceae	*Alnus glutinosa* L.	Alksnis	L		5	Bark	Therapeutic bath	To reduce skin inflammation	
Betulaceae	*Betula pendula* Roth.	Beržas	K<br>L	16	10<br>2<br>2	Aerial parts<br><br>Buds	Infusion<br>Therapeutic bath<br>Decoction	Wound healing<br><br>To treat dermatitis	EMA/HMPC/573241/2014

					12<br><br>7<br>6<br>4	Buds<br><br>Leaves<br>Leaves<br>Leaves	Decoction<br>Decoction<br>Decoction	To treat skin rashes and acne<br>For skin lightening<br>To strengthen hair<br>From hair loss	Betulae folium; Urinary tract disorders
Boraginaceae	*Symphytum officinale L.*	Vaistinė taukė<br>Kaulažolė	L<br>K	18	5<br>3<br>14<br>11	Roots<br>Roots<br>Roots<br>Roots	Decoction<br>Ointment<br>Ointment<br>Ointment	Wound healing<br>For cracked heels<br>For joint pain<br>For bruises	EMA/HMPC/572846/2009 *Symphyti radix;* Symptomatic treatment of bruises and sprains
Brassicaceae	*Armoracia rusticana*	Valgomasis krienas	L	7	7	Leaves	Topical application	For bruises	
Brassicaceae	*Brassica oleracea* L.	Kopūstas<br>Kopūsts	L	24	10<br><br>20<br><br>14	Leaves<br><br>Leaves<br><br>Leaves	Topical application<br>Topical application<br>Topical application	For furuncles and abscesses<br>To reduce skin inflammation<br>For joint pain	
Cannabaceae	*Humulus lupulus* L.	Apyniai	K	1	1	Aerial parts	Decoction	To strengthen hair	EMA/HMPC/682384/2013 *Lupuli flos;* Stress and sleep disorders
Caryophyllaceae	*Saponaria officinalis*	Muiliažolė	K	19	19	Aerial parts	Juice	For skin cleansing	
Caryophyllaceae	*Stellaria media* (L.) Vill.	Žliūgė	L	8	4<br><br>4	Aerial parts<br><br>Leaves	Infusion<br><br>Therapeutic bath	To treat skin rashes and acne<br>For bruises	
Commelinaceae	*Callisia fragrans*	Auksinis ūsas	L	5	5	Leaves	Compress	For bruises	
Crassulaceae	*Crassula ovata*	Storlapis	L	1	1<br><br>1	Leaves<br><br>Leaves	Topical application	Wound healing<br><br>To treat calluses	
Crassulaceae	*Kalanchoe daigremontiana*	Kalankė, Paleistuvė	L	6	4<br>4<br>3	Leaves	Compress<br>Tincture<br>Juice	For bruises<br><br>To treat calluses	

Cucurbitaceae	*Cucumis sativus*	Paprastasis agurkas	K	18	17<br><br>7<br><br>5	Fruits<br><br>Fruits<br><br>Fruits	Puree (as facial mask)<br>Puree (as facial mask)<br>Puree (as facial mask)	For dry skin<br>For dark spots<br>To reduce under-eye puffiness	
Elaeagnaceae	*Hippophae rhamnoides L.*	Šaltalankiai	L<br>K	18	18<br>1<br>9<br>3<br>8<br><br>8	Seeds	Oil	Wound healing<br>To treat bedsores<br>For burns<br>For dry skin<br>To reduce skin inflammation<br>To reduce itching	
Equisetaceae	*Equisetum arvense*	Dirvinis asiūklis	L	3	3	Aerial parts	Decoction (rinse)	Wound healing	EMA/HMPC/278091/2015 *Equiseti herba;* Urinary tract disorders; Skin disorders and minor wounds
Ericaceae	*Calluna vulgaris* (L.) Hull.	Šilinis viržis	L	1	1<br><br>1	Flores	Topical application	Wound healing<br><br>To treat bedsores	
Fabaceae	*Trifolium repens L.*	Balti dobiliukai	K	6	6	Aerial parts	Therapeutic bath	To reduce skin inflammation	
Fagaceae	*Quercus robur*	Ąžuolas	KL	17	9<br>7<br><br>4<br><br>6<br><br>9	Bark<br>Bark<br><br>Bark<br><br>Bark<br><br>Bark	Decoction<br>Decoction<br><br>Therapeutic bath<br>Decoction<br><br>Decoction	Wound healing<br>To treat skin rashes and acne<br>To reduce (allergic) itching<br>To reduce enlarged pores<br>For hair darkening	EMA/HMPC/3203/2009 *Quercus cortex;* Mild diarrhoea; minor inflammation of oral mucosa and skin; To relieve of haemorrhoidal symptoms
Geraniaceae	*Pelargonium odoratissimum*	Jeronimas Pelargonija	L	17	17	Leaves	Topical application	To treat ear pain and inflammation	
Grossulariaceae	*Ribes nigrum L.*	Juodųjų serbentų	L	3	3	Leaves	Decoction	Wound healing	EMA/HMPC/745353/2016 *Ribis nigri folium;* Joint pain and minor inflammation; Urinary tract flushing

Grossulariaceae	*Ribes uva-crispa* L.	Agrastas	K	1	1	Fruits	Puree	For skin lightening	
Hypericaceae	*Hypericum perforatum L.*	Jonažolė	L<br>K	15	2<br>5<br>4<br>10<br><br>1<br>1<br>1<br>1	Aerial parts	Hydrolate<br>Infusion<br>Infusion<br>Infusion (rinse)<br>Hydrolate<br>Hydrolate<br>Hydrolate<br>Hydrolate<br>Compress	To reduce skin inflammation<br>For burns<br>Wound healing<br><br>From hair loss<br>For wrinkles<br>To reduce itching<br>For wart treatment	EMA/HMPC/7695/2021 *Hyperici herba;* Gastrointestinal disorders; Skin disorders and minor wounds; Mental stress and mood disorders
Juglandaceae	*Juglans regia* L.	Riešutmedis	L<br>K	7	7	Leaves	Decoction	Wound healing	EMA/HMPC/346737/2011 *Juglandis folium;* Minor skin inflammations; excessive sweating of the hands and feet
Lamiaceae	*Glechoma hederacea* L.	Tramažolė	L	4	4<br>4	Leaves	Topical application	From insect bites<br>For bruise	
Lamiaceae	*Hyssopus officinalis*	Juozažolė<br>Isopas	L	1	1<br>1	Flores<br>Flores	Hydrolate<br>Hydrolate	From insect bites<br>To reduce itching	
Lamiaceae	*Lavandula angustifolia*	Levanda	K	6	6<br>6	Flores<br>Flores	Hydrolate<br>Hydrolate	For dry skin<br>To reduce skin inflammation	EMA/HMPC/734125/2010 *Lavandulae flos;* Mental stress and sleep disorders
Lamiaceae	*Melissa officinalis* L.	Melisa	L	3	3	Leaves	Infusion	To reduce skin inflammation	EMA/HMPC/196745/2012 *Melissae folium;* Gastrointestinal disorders; Mental stress and sleep disorders
Lamiaceae	*Mentha piperita L.*	Mėta	L<br>K	8	3<br>3<br><br>6	Aerial parts / Leaves<br><br>Aerial parts	Hydrolate /Topical application<br>Hydrolate	From insect bites<br>To reduce itching<br><br>For oily skin	*EMA/HMPC/572705/2014 Menthae piperitae folium;* Gastrointestinal disorders
Lamiaceae	*Nepeta cataria* L.	Katžolė<br>Nepeta	L		1	Leaves	Infusion	From insect bites	
Lamiaceae	*Ocimum basilicum*	Bazilikas	L<br>K	4	3	Leaves	Topical application	From insect bites	

					2	Leaves	Infusion	For cleansing oily skin	
Lamiaceae	*Origanum vulgare*	Raudonėlis	L<br>K	6	6<br><br>6<br>2<br>1	Aerial parts	Hydrolate<br><br>Decoction<br>Hydrolate<br>Hydrolate	To treat skin rashes and acne<br>Wound healing<br>From insect bites<br>To treat cold sores	
Lamiaceae	*Salvia officinalis* L.	Vaistinis šalavijas	L	12	10<br>2	Aerial parts	Infusion<br>Hydrolate	To reduce skin inflammation	EMA/HMPC/277152/2015 *Salviae officinalis folium;* Gastrointestinal disorders; Excessive sweating; Skin disorders and minor wounds; Mouth and throat disorders
Lamiaceae	*Thymus vulgaris*	Čiobrelis	L	4	4<br>4	Aerial parts	Hydrolate	From insect bites<br>Wound healing	EMA/HMPC/342332/2013 *Thymi herba;* Cough and common cold
Linaceae	*Linum usitatissimum* L.	Linas	L	7	7<br>7	Seeds<br>Seeds	Decoction<br>Decoction	To treat dermatitis<br>For dry skin	EMA/HMPC/377675/2014 *Lini semen;* Gastrointestinal disorders
Malvaceae	*Althaea officinalis*	Svilarožė	L	4	4<br>4	Roots<br>Roots	Decoctio<br>Decoction	Wound healing<br>For sunburn	EMA/HMPC/436679/2015 *Althaeae radix;* Gastrointest-inal disorders; Irritation of oral mucosa and dry cough
Oleaceae	*Syringa vulgaris* L.	Alyva	L	2	2	Leaves	Infusion	Wound healing	
Paeoniaceae	*Paeonia lactiflora*	Bijūnas	L<br>K	3	3<br><br>3	Flores<br><br>Flores	Hydrolate<br><br>Hydrolate	To reduce skin inflammation<br>For dry skin	
Papaveraceae	*Chelidonium majus* L.	Ugniažolė<br>Karpažolė	L	31	31<br>2<br>1	Stem<br>Aerial parts	Juice<br>Hydrolate<br>Hydrolate	For wart treatment<br>Wound healing<br>For intertrigo	
Pinaceae	*Pinus sylvestris* L.	Pušis<br>Pušelė	K	3	3	Buds	Decoction	For oily skin	
Plantaginaceae	*Plantago major* L.	Trauklapis<br>Gyslotis	L	35	35<br>8<br>2	Leaves<br>Leaves<br>Aerial parts	Topical application<br>Ointment	For bruises<br>Wound healing	

					10	Leaves	Compress	For furuncles and abscesses	
Poaceae	*Avena sativa* L.	Aviža<br>Sėjamoji aviža	L<br>K	6	6<br>6<br>6	Fruits<br>Fruits<br>Fruits	Therapeutic bath<br>Decoction	For sunburns<br>To reduce itching<br>For dry skin	EMA/HMPC/368600/2007<br>*Avenae fructus; Minor* skin inflammation and minor wounds<br>EMA/HMPC/202966/2007<br>*Avenae herba;* Mild mental stress and sleep disorders
Polygonaceae	*Polygonum aviculare L.*	Rūgtis<br>Takažolė	L	1	1	Aerial parts	Decoction	Wound healing	EMA/HMPC/143658/2015<br>*Polygoni avicularis herba;*Common cold; Minor inflammation of oral mucosa; Urinary tract flushing
Primulaceae	*Primula veris* L.	Pavasarinė raktažolė	K	1	1<br>1	Flores<br>Leaves	Infusion<br>Infusion	For dark spots (rinse face)<br>For wrinkles	EMA/HMPC/136582/2012<br>*Primulae flos;* Common cold and cough<br>EMA/HMPC/104095/2012<br>*Primulae radix;* Common cold and cough
Rosaceae	*Alchemilla vulgaris* L.	Šlaitinė rasakila	L	1	1	Leaves	Therapeutic bath	To treat skin rashes and acne	
Rosaceae	*Filipendula ulmaria (L.) Maxim.*	Vingiorykštė	L	8	1<br>7	Roots<br>Aerial parts	Ointment<br>Decoction	To treat eczema<br>To reduce skin inflammation	EMA/HMPC/434894/2010<br>*Filipendulae ulmariae flos;* Common cold symptoms; Minor articular pain<br>EMA/HMPC/434881/2010<br>*Filipendulae ulmariae herba;* Common cold symptoms; Minor articular pain
Rosaceae	*Fragaria vesca* L.	Žemuogė	K	3	3<br>3	Fruits<br>Fruits	Puree (as facial mask)	For skin lightening<br>For dark spots	EMA/HMPC/432278/2015<br>*Fragariae folium;* Urinary tract flushing; Mild diarrhoea
Rosaceae	*Fragaria x ananassa*	Braškė	K	4	4	Fruits	Puree (as facial mask)	For dark spots	EMA/HMPC/432278/2015

									Fragariae folium; Urinary tract flushing; Mild diarrhoea
Rosaceae	*Rosa damascena* Mill.	Rožė<br>Erškėtis	K	11	6<br>5	Flores<br>Flores	Hydrolate<br>Hydrolate	For dry skin<br>For oily skin	EMA/HMPC/137299/2013<br>*Rosae flos;* skin and mucosal inflammation
Rosaceae	*Sorbus aucuparia* L.	Šermukšnis	L	6	6	Bark	Decoction<br>Therapeutic bath	Wound healing	
Salicaceae	*Populus nigra*	Topolis	L	6	6	Buds	Tincture	Wound healing<br>For rinsing wound	
Solanaceae	*Solanum lycopersicum L.*	Pomidoras	K	3	3	Fruits	Juice	For dry, cracked heels	
Solanaceae	*Solanum tuberosum* L.	Bulvė	L<br>K	19	18<br>3	Tubers<br>Tubers	Puree<br>Puree (as facial mask)	For burns<br>To reduce under-eye puffiness	
Tropaeolaceae	*Tropaeolum majus* L.	Nasturta	L	12	7<br><br>5<br><br>2<br>2	Leaves<br><br>Leaves<br><br>Flores<br>Flores	Topical application<br><br><br>Decoction<br>Decoction	To reduce skin inflammation<br>For furuncles and abscesses<br>To reduce dandruff<br>To strengthen hair	
Urticaceae	*Urtica dioica* L.	Didžioji dilgelė	K<br>L	30	16<br>4<br>20<br>15<br>8<br>4	Aerial parts<br><br>Aerial parts<br>Aerial parts<br>Aerial parts<br>Aerial parts	Decoction<br>Hydrolate<br>Decoction<br>Decoction<br>Decoction<br>Decoction	From hair loss<br><br>To strengthen hair<br>To reduce dandruff<br>To treat dermatitis<br>For hair darkening	EMA/HMPC/ 261302/2022<br>*Urticae herba;* Urinary tract disorders; joint pain; skin disorders, seborrheic skin
Violaceae	*Viola tricolor* L.	Našlaitė	L	4	3<br>1	Aerial parts<br>Flores	Decoction	To treat skin rashes and acne<br>To treat dermatitis	EMA/HMPC/131734/2009<br>*Violae herba cum flore;* Skin disorders, seborrheic skin
